# The Impact of Nutritional Supplementation on Sweat Metabolomic Content: A Proof-of-Concept Study

**DOI:** 10.3389/fchem.2021.659583

**Published:** 2021-05-07

**Authors:** Sean W. Harshman, Andrew B. Browder, Christina N. Davidson, Rhonda L. Pitsch, Kraig E. Strayer, Nicole M. Schaeublin, Mandy S. Phelps, Maegan L. O'Connor, Nicholas S. Mackowski, Kristyn N. Barrett, Jason J. Eckerle, Adam J. Strang, Jennifer A. Martin

**Affiliations:** ^1^UES Inc., Air Force Research Laboratory, 711th Human Performance Wing/RHBBF, Wright-Patterson AFB, Dayton, OH, United States; ^2^Air Force Research Laboratory, 711th Human Performance Wing/RHBBF, Wright-Patterson AFB, Dayton, OH, United States; ^3^InfoSciTex Corp., Air Force Research Laboratory, 711th Human Performance Wing/RHBCN, Wright-Patterson AFB, Dayton, OH, United States; ^4^Air Force Research Laboratory, 711th Human Performance Wing/RHBCN, Wright-Patterson AFB, Dayton, OH, United States

**Keywords:** sweat, metabolomics, diet, quantitation, normalization

## Abstract

Sweat is emerging as a prominent biosource for real-time human performance monitoring applications. Although promising, sources of variability must be identified to truly utilize sweat for biomarker applications. In this proof-of-concept study, a targeted metabolomics method was applied to sweat collected from the forearms of participants in a 12-week exercise program who ingested either low or high nutritional supplementation twice daily. The data establish the use of dried powder mass as a method for metabolomic data normalization from sweat samples. Additionally, the results support the hypothesis that ingestion of regular nutritional supplementation semi-quantitatively impact the sweat metabolome. For example, a receiver operating characteristic (ROC) curve of relative normalized metabolite quantities show an area under the curve of 0.82 suggesting the sweat metabolome can moderately predict if an individual is taking nutritional supplementation. Finally, a significant correlation between physical performance and the sweat metabolome are established. For instance, the data illustrate that by utilizing multiple linear regression modeling approaches, sweat metabolite quantities can predict VO_2_ max (*p* = 0.0346), peak lower body Windage (*p* = 0.0112), and abdominal circumference (*p* = 0.0425). The results illustrate the need to account for dietary nutrition in biomarker discovery applications involving sweat as a biosource.

## Introduction

As wearable sensors, such as smart watches, become more socially integrated, the need for novel biosources to provide real time feedback of performance is paramount. The non-invasive, on-demand, real-time characteristics of sweat make it an ideal biomedia for this application. Although analyzed for several decades, more recent advances in analytical tools, allowing for a greater depth of analysis, and the progress of microfluidic wearable sensor technology have thrust sweat into the forefront of biomarker discovery efforts (Robinson and Robinson, 1954). However, to achieve the goal of continuous on human monitoring via wearable sensors utilizing sweat, many attributes of sweat must be examined, such as analyte selection, the analyte concentration, and the impact of factors affecting variability, among many other considerations.

Advances in mass spectrometry (MS) and nuclear magnetic resonance (NMR) instrumentation and methodologies have allowed for a movement from cation and anion monitoring to a more discovery-based approach to sweat analysis (Kutyshenko et al., 2011; Calderón-Santiago et al., 2014; Mena-Bravo and Castro, 2014; Delgado-Povedano M. M. et al., 2016). As a result, a greater understanding of the sweat analyte content has emerged. For instance, evidence suggests sweat contains measurable quantities of proteins and metabolites, in addition to the previously mentioned electrolytes, while the data surrounding the sweat lipid content is still emerging (Patterson et al., 2000; Agrawal et al., 2017, 2018; Yu et al., 2017; Delgado-Povedano et al., 2018). Although sweat proteomic analysis has struggled due to low sweat protein abundance, high salt content, and an observation of a large quantity of non-specific peptides, sweat metabolomics has proven more favorable for analyte identification and preliminary biomarker applications (Calderón-Santiago et al., 2014; Delgado-Povedano M. del M. et al., 2016; Macedo et al., 2017; Yu et al., 2017; Delgado-Povedano et al., 2018; Harshman et al., 2018, 2021). For example, the sweat metabolome has been primarily defined by amino acids and amino acid like compounds with connections of metabolite abundance to health and disease (Delgado-Povedano M. M. et al., 2016; Macedo et al., 2017; Delgado-Povedano et al., 2018; Harshman et al., 2018). However, inherent difficulties exist in metabolite compound identification, such as adducts and dimerization, access to neat standards, competing background ions, instrument noise, co-elution of isomeric species, source fragmentation, and the relatively simplistic nature of metabolites (Xu et al., 2015; Domingo-Almenara et al., 2017; Cho et al., 2021). Furthermore, MS detector speed can limit the type of quantitative and/or qualitative data to be collected, guiding the experiment to be targeted, untargeted, or semi-targeted in a blended data dependent acquisition (DDA) approach (Cho et al., 2021). Because of these intrinsic conditions, novel sweat metabolite identifications have remained sparce. For instance, a recent sweat metabolomic discovery experiment confirmed the identity of 40 metabolomic compounds, of which nine were previously unidentified by other groups (Harshman et al., 2018). Furthermore, data indicate expression of many intercorrelated metabolites within sweat suggesting compound adducts could be prevalent (Harshman et al., 2018, 2021). As a result of the lack in complexity among sweat content and the link between metabolite abundance and performance, the need to move from relative quantitation toward absolute quantitation is necessary to achieve the goal of successful biomarker discovery and real-time sensor design.

While many quantitative ranges have been defined for ions, such as sodium and potassium, in sweat, the metabolomic quantitation has lacked (Brusilow and Gordes, 1968; Fukumoto et al., 1988; Patterson et al., 2000, 2002; Morgan et al., 2004; Alvear-Ordenes et al., 2005; Meyer et al., 2007; Sakharov et al., 2010; Harshman et al., 2018). For instance, recent data suggest amino acid quantities in sweat have been shown to range from ~360 to <5 μM with alanine illustrating the greatest overall amount (Harshman et al., 2019) To advance the potential for sweat to be used for biomarker discovery and ultimately transition to real-time sensor development, additional metabolites must be quantitated and sources of variability must be determined to truly understand the sweat metabolome dynamics.

Although many sources of variability could contribute to sweat dynamics, prominent sources include diet and sweat rate. The link between diet and altered sweat content has been established although the metabolites investigated were limited. For example, Patterson et al. illustrated sweat pH could be increased by ingesting ${\text{NaHCO}}_{3}^{-}$ (Patterson et al., 2002). Additionally, Czarnowski et al. showed sweat ammonia concentrations could be affected by a low carbohydrate diet (Czarnowski et al., 1995). The remaining research surrounding the link between sweat and diet has focused on electrolytes and minerals such as Na, Zn, Fe, Ca, and Cu (Baker, 2019). Furthermore, the impact of localized sweat rate normalization on the sweat metabolite abundance was recently shown to increase variability among individuals sampled (Harshman et al., 2021). However, the normalization strategy determined by Harshman et al. is not applicable to a bulk patch collection. Therefore, additional experimentation is required to expand the link between small molecule metabolites and nutrition, in addition to identifying an alternative method for data normalization from patch collected sweat.

Here, a targeted metabolomics approach is applied to sweat collected from participants in an exercise program ingesting either a low or high nutritional supplementation. Using the dried powdered mass of the sweat for normalization, the data support the hypothesis that ingestion of dietary supplementation can quantitatively affect the sweat metabolome. Furthermore, correlation between physical performance, nutritional supplementation, and the sweat metabolome are established. The results illustrate the need to account for nutrition in biomarker discovery applications involving sweat as a biosource.

## Experimental

### Human Subjects

All volunteer human subjects (*n* = 13) were male members of the United States Air Force of variable age (19–35) and rank stationed at Wright-Patterson Air Force Base (AFB), Ohio, USA for this proof-of-concept study. Permission to perform human subjects research was obtained, prior to the study's start, from the Wright-Patterson AFB Institutional Review Board (IRB# FWR20150032H). Volunteers were informed of the protocol and permitted to ask questions, then provided informed written consent to participate.

### Sweat Stimulation and Sample Collection

**Figure 1:**
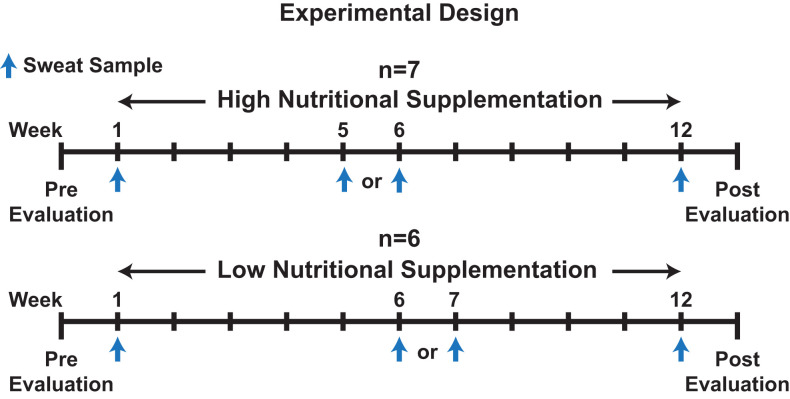
An illustration depicting the experimental design for the overall experiment and sweat collection time points.

Participants' sweat was sampled during an ongoing physical fitness and nutritional intervention study within the Air Force Research Laboratory (Zwilling et al., 2020). Prior to (Pre) and following (Post) the 12-week fitness program, physical performance was evaluated by measuring weight, VO_2_ max, resting heart rate (HR), abdominal circumference (Ab Circ), % body fat, push-ups, sit-ups, and upper body (UBW) & lower body Wingate (LBW) testing as outlined by Zwilling et al. (2020). Additionally, as part of the experiment by Zwilling et al., volunteers were provided a twice daily (7 days a week for 12 weeks), orally ingested, liquid high nutritional dietary supplementation (*n* = 7) or low nutritional supplementation (*n* = 6) drink of which subjects were blinded (Zwilling et al., 2020). No traditional placebo group was utilized. Finally, study participants performed programmed daily (Monday through Friday) strength training and/or cardiovascular exercise routines over the 12-week period. Please refer to Figure 1 and Supplementary Data 1, 2, 3 for additional information regarding the experiment design, physical performance evaluation results, and participant exercise information, and to Zwilling et al. (2020) for the composition of the two supplementations.

Sweat was collected from forearms of participants performing their routine cardio workout on either a stationary bike, elliptical, or treadmill, as part of the larger Zwilling et al. experiment, on week 1, week 5, 6, or 7, and week 12 (Figure 1) (Zwilling et al., 2020). Week 5, 6, or 7 was used as a mid-point sweat collection as participant attendance varied and was not 100% amongst all participants throughout the study. Depending on the week the participant missed, either week 5, 6, or 7 was utilized as the mid-point. The temperature and humidity (% RH) within the exercise laboratory was measured during collection with a Kestrel 4500NV weather tracker (Temperature: 22.2 ± 1.3°C, % RH: 0.2%, Supplementary Data 4). Percent max heart rate (% maxHR), determined during a prior VO_2_ max test was utilized to regulate intensity among workouts (Parvo Medics, Sandy, UT, USA). Workouts consisted of periods of variable %maxHR over a 22-min exercise program. Please refer to Supplementary Data 3 for a summary of the exercise protocols, exercises performed, and training loads from exercises.

Sweat was collected via a forearm patch, as described previously (Brisson et al., 1991; Harshman et al., 2019). Briefly, both forearms at the inner side of the arm between the elbow and wrist with minimal hair were wiped thoroughly with a brand new 2-propanol wipe (BD Biosciences, San Jose, CA, USA). Following, each forearm was rinsed for 5–10 s with tap water and allowed to air dry. As described in Harshman et al., a modified patch was applied to the center, estimated as the hairless area approximately at the middle between the elbow and wrist, of the dried forearm (Harshman et al., 2019).

Immediately at the conclusion of the exercise, free sweat within the patch was aspirated with a 5 mL syringe and needle, blunt tipped (needle and syringe, Hamilton, Reno, NV, USA). The aspirate was placed in the top of a 0.1 μm polyvinylidene difluoride (PVDF) centrifugal filter, one filter per arm (Ultrafree-CL PVDF filter, Millipore, Burlington, MA, USA). Additionally, the nylon piece from the collection pouch was added to the corresponding arm's centrifugal filter, using forceps, and both filters were spun at 3,000 × g for 10 min. The filtrates, from both arms were pooled together. A 250 μL aliquot of the combined filtered sweat was placed into a dried, preweighed lo-bind Eppendorf tube and frozen on liquid nitrogen (balance, Mettler Toledo, Columbus, Ohio, USA, Eppendorf, Hamburg, Germany). Aliquots were lyophilized to dryness, reweighed, and stored at −80°C until use (Labconco, Kansas City, MO, USA). The mass of the dried sweat was determined by subtracting the dried sweat mass from the dried empty tube mass. Please refer to Supplementary Data 5 for the determined dried sweat masses.

### Sweat Metabolomics Sample Preparation

All metabolomics samples were reconstituted in 250 μL of 50% acetonitrile (aq) supplemented with 30 μM of each isotopically labeled standard: taurine, choline, creatinine, citrulline, pyrrolidine, alanine, arginine, glutamic acid, histidine, isoleucine, leucine, lysine, methionine, phenylalanine, proline, tyrosine, and valine (Resuspension Buffer, solvents: Optima Grade, ThermoFisher Scientific, Waltham, MA, USA). Refer to Supplementary Data 6 for information regarding the isotopic label, manufacturer, and purity. Samples were thoroughly mixed. Ten microliters of each sample were removed, combined to create a pooled sample, and thoroughly mixed. The samples and pooled sample were transferred to vials for liquid chromatography mass spectrometry (LC-MS) analysis. All samples were run in a random order determined within the Microsoft Excel program using the RANDBETWEEN function (Redmond, WA, USA). Pooled samples were analyzed after every 10 unknown sweat samples with blank injections [50% acetonitrile (aq)] run after every sweat sample and pooled sample.

### Liquid Chromatography-Mass Spectrometry (LC-MS)

All sweat samples, pooled samples, standard curves, and blanks (2 μL injections) were separated via hydrophilic interaction liquid chromatography (HILIC) on a Vanquish Horizon UPLC system paired with high resolution accurate mass (HRAM) detection on a Q-Exactive HF mass spectrometer (Thermo Fisher Scientific). Separations were performed at 170 μL min^−1^ using a Waters Acquity BEH Amide column (130 Å, 1.7 μm, 2.1 × 100 mm) with mobile phase A consisting of 10 mM ammonium formate in 4.5% acetonitrile (aq) and mobile phase B consisting of 10 mM ammonium formate in 95.5% acetonitrile (aq) (MS grade, ≥99.0%, Sigma Aldrich, St. Louis, MO, USA). A 30-min gradient of mobile phases was run, beginning with 90% mobile phase B for 5 min. The percent B transitioned to 65% at 18.5 min and held at 65% for 5 min (23.5 min). The gradient returned to 90% B at 24.5 min and remained at 90% B for the remainder of the run. The mass spectrometer source was operated in positive ionization mode with 3.5 kV spray voltage, 250°C capillary temperature, and sheath gas of 30. The intact mass scans (MS1 only) were acquired at 60,000 resolution over a 65–400 m/z range (profile) with a 1 × 10^6^ AGC target and a 50 ms maximum ion accumulation time.

### Sweat Metabolomics Calibration

Calibration curves for 23 compounds previously identified in human sweat, were generated in Resuspension Buffer across various ranges (5 and 300 μM) (Liappis et al., 1979; Calderón-Santiago et al., 2014, 2015; Dutkiewicz et al., 2014; Hooton et al., 2016; Macedo et al., 2017; Delgado-Povedano et al., 2018; Harshman et al., 2018, 2019). Calibration curves were analyzed, in increasing concentration, by LC-MS at the beginning, middle, and end of the overall sample analyses, as described in the following section. Peak areas and retention times for each compound, including the isotopically labeled compounds, were determined using the Tracefinder EFS software package (v. 3.2, Thermo Fisher Scientific). Calibration curves were created for each individual compound by plotting theoretical concentration, in μM, on the x-axis and the unlabeled/isotopically labeled peak area ratio of all calibrant injections on the y-axis. A line of best fit was fit to the data and an equation of the line was generated (Prism Graphpad Software, v. 9.0, La Jolla, CA, USA). For those compounds where an isotopically labeled version of the compound were unavailable, raw peak areas of all calibrant injections were plotted on the y-axis rather than the unlabeled/isotopically labeled peak area ratios. Please refer to Supplementary Data 6 for individual compound: quantitative ion, calibration ranges, line of best fit equation, and *R*^2^ value of the best fit line.

### Metabolite Semi-Quantitation

Peak areas and retention times for pooled samples and unknown sweat samples were determined using the Tracefinder EFS software and quantitative ions as described for the calibration standards. The semi-quantitative values for each unknown and pooled sample injection were determined by inputting the unlabeled/isotopically labeled peak area ratio or peak area for those compounds without an isotopically labeled compound pair into the equation (y) of the line of best fit for each individual compound and solving for x (Supplementary Data 7). All processed data have been provided as Supplementary Data 8.

### Statistical Analysis

Basic statistical analysis was performed within the Prism Graphpad Software Suite (v. 9.0.0, LaJolla, CA, USA). All additional statistical analyses, including calculation of the log_2_ fold change utilized to evaluate proportional changes in the data, were performed using RStudio software suite (v. 3.6.3) within the R statistical platform (v. 1.2.1335, Boston, MA, USA, (R Computing Team, 2020). PCA plots and heatmaps were illustrated using “gplots” and “ggbiplot” packages (Vu; Warnes et al., 2020).

## Results

### Sweat Metabolite Semi-Quantitation

Excreted sweat has primarily been a matrix investigated with respect to cation and anion analysis (Morimoto and Johnson, 1967; Brusilow and Gordes, 1968; Allan and Wilson, 1971; Fukumoto et al., 1988; Falk et al., 1991; Shirreffs and Maughan, 1997; Patterson et al., 2000, 2002; Hayden et al., 2004; Morgan et al., 2004; Saat et al., 2005; Buono et al., 2007; Meyer et al., 2007; Baker et al., 2009; Harshman et al., 2021). More recently mass spectrometry approaches have been applied to sweat to further investigate the other non-volatile components (Calderón-Santiago et al., 2014; Delgado-Povedano M. del M. et al., 2016; Hooton et al., 2016; Macedo et al., 2017; Delgado-Povedano et al., 2018; Harshman et al., 2018, 2019, 2021). However, quantitation of discovered sweat metabolites has remained sparce (Harshman et al., 2019). To further establish semi-quantitative values associated with sweat metabolite abundance, calibration curves for 23 previously identified compounds were generated (Supplementary Data 7). Determination of the unknown peak areas illustrated that 15 of the 23 compounds (creatinine, phenylalanine, leucine, isoleucine, methionine, valine, proline, tyrosine, alanine, glutamic acid, citrulline, histidine, arginine, lysine, ornithine) had abundances consistently within the detectable and calibrated ranges. The remaining compounds, pyrrolidine, choline, dimethanolamine, prolinamide, trolamine, diolamine, carnitine, and taurine, were consistently quantified below the calibrated range (<5 μM) and were removed from the analysis. Interestingly, while preliminary results and previous data suggested the upper limit of the calibrated range (300 μM) would be sufficient to encompass all sweat samples, six of the 15 compounds (creatinine, alanine, citrulline, histidine, arginine, and ornithine) have unknown concentrations greater than the calibrated range (Supplementary Data 9A) (Harshman et al., 2019). Conversely, a single compound, isoleucine, had a single value below the lower limit of the calibration (5 μM). Due to the unanticipated large dynamic range of several unknown sample concentrations, the values were included in the analysis but considered estimations based on extrapolating the line of best fit. The results illustrate a large dynamic range for several compounds among individual sweat samples.

To evaluate instrument and sample stability over the entire analysis, the mean area, standard deviation, and % relative standard deviation (%RSD) of each compound in the pooled sample was tabulated and provided in Supplementary Data 9B. Of note, a large pooled sample %RSD values were observed for methionine and ornithine (>10%). Upon manual inspection of the raw data, the result can be attributed to retention time variability and/or coelution of similar masses at retention times close to that of methionine and ornithine in two of the eight pooled sample injections. The two injections in question were in the middle of the analytical run suggesting the variability was not related to sample degradation or instrument instability. As a result, the large pooled sample %RSD for methionine and ornithine were noted but the compounds were included in the overall analysis. Conversely, the remaining compounds from the pooled samples illustrate overall low variability (<10% RSD) for all eight injections (Supplementary Data 9B). Collectively, these data suggest analytical stability for the entire semi-quantitative analysis.

### Normalization of Sweat Metabolite Abundance to Dried Powder Mass

**Figure 2:**
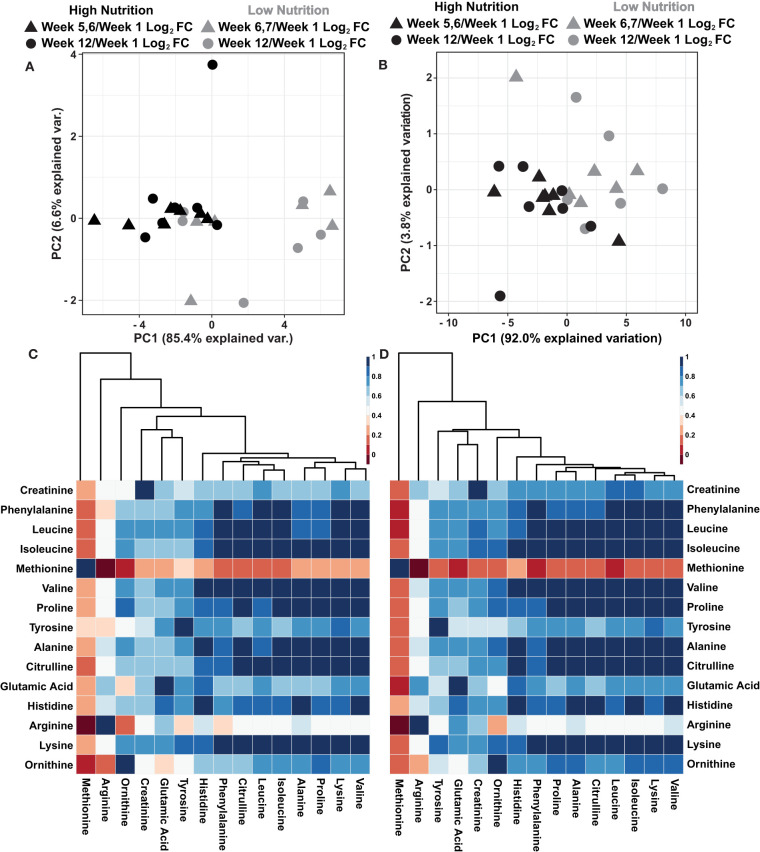
**(A)** An autoscaled and mean centered PCA of the non-normalized metabolite log_2_ fold change, relative to week 1, parsed by nutritional supplementation. **(B)** An autoscaled and mean centered PCA of the dried sweat mass normalized metabolite log_2_ fold change values, relative to week 1, parsed by nutritional supplementation. **(C)** A cluster map of the calculated Pearson correlation coefficients of non-normalized metabolite values. **(D)** A cluster map of the calculated Pearson correlation coefficients of dried sweat mass normalized metabolite values. Cluster maps utilize Euclidean distance and average linkages. The data illustrate greater explained variability and higher intercorrelation of metabolite data when normalized to the dried powder mass of sweat.

A recent report suggests that sweat rate normalization significantly influenced sweat global metabolomic data (Harshman et al., 2021). While the collection methodologies as described by Harshman et al. (2021) were unavailable at the time of sampling to determine localized sweat rate, the dried powdered mass of sweat was determined for each 250 μL sweat sample following lyophilization (Supplementary Data 5). To evaluate the utility of normalizing sweat metabolomics data with the dried powder mass, two principal component analyses (PCA) were performed using log_2_ fold change metabolite values relative to week 1 and either non-normalized or normalized to the dried powder mass of the sweat (Figures 2A,B). The results illustrate the dried powder mass normalized data has greater explained variation within the first two principal components (95.8%) compared to the non-normalized data (92.0%). Furthermore, the dried powder mass normalized data show greater spread among the data points particularly a within the high nutritional supplement group (Figures 2A,B). Previous results indicated a high amount of intercorrelation among sweat metabolite abundances with increased correlation following rate normalization (Harshman et al., 2018, 2021) To evaluate if similar trends exist within these data, Pearson correlation coefficients were determined among the log_2_ relative metabolite abundance of non-normalized and dried powder mass normalized data (Figures 2C,D). The results indicate the dried powder mass normalized data has a higher amount of intercorrelation among the metabolite abundances compared to the non-normalized values. Collectively, these results indicate a greater variability among the data is accounted for with dried powder mass normalization in line with previous observations surrounding sweat data normalization (Harshman et al., 2021). However, further research and validation surrounding this data normalization is needed.

### Sweat Metabolite Abundance and Nutritional Supplementation

**Figure 3:**
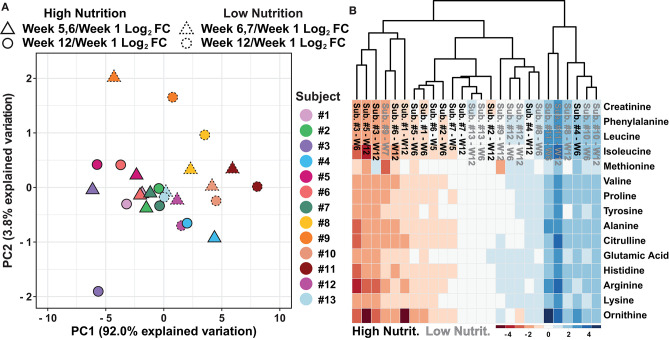
**(A)** An autoscaled and mean centered PCA of the dried sweat mass normalized log_2_ fold change, relative to week 1, parsed by nutritional supplementation and individual subjects. **(B)** A cluster map of the dried sweat mass normalized log_2_ fold change values, relative to week 1, for 15 measured metabolites in sweat by individual and nutritional supplementation. W indicates week and Cluster map utilize Euclidean distance and average linkages. The data suggest relative sweat metabolite abundance can separate individuals based on high or low nutritional supplementation.

To determine if the data separate based on the dietary supplement group, further evaluation of the PCA shown in Figure 2B was conducted. The results suggest the data separate based on the nutritional supplementation provided to the individual, high (black) and low (gray) (Figure 2B). Evaluation of the variable biplot and PCA loadings indicate all variables contribute equally to PC1 except methionine which contributes primarily to PC2 (Supplementary Data 10). Furthermore, as individual samples within the PCA are labeled, the data show individual's week 5, 6, or 7 and week 12 log_2_ fold change data, relative to week 1, are in close proximity with data points of the same color, suggesting small amounts of change from the middle samples (week 5, 6, or 7) to the end samples (week 12, Figure 3A). To evaluate how the metabolomics data are influenced by high and low nutritional supplementation, a cluster map of the mass normalized log_2_ fold change, relative to week 1, was constructed (Figure 3B). The results show the individuals provided the high nutritional supplement had lower quantities of the measured metabolites, relative to week 1, when compared to those given the low nutritional supplement. Collectively, the data support the hypothesis that the sweat metabolome can be impacted by an individual's nutritional intake.

### Sweat Metabolomics, Nutritional Supplementation, and Human Performance

**Figure 4:**
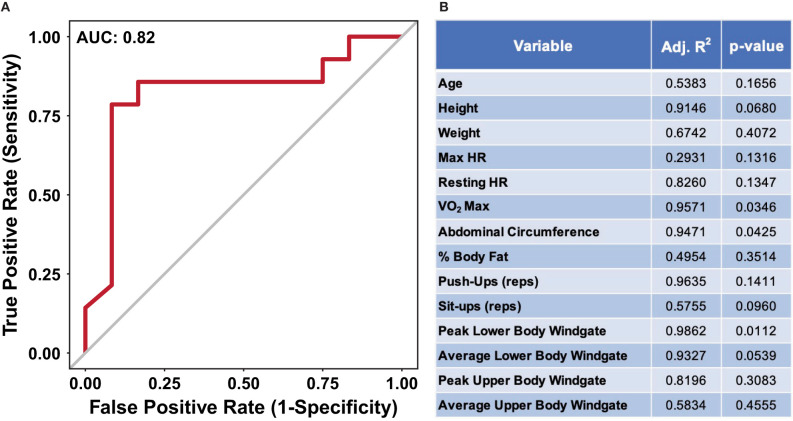
**(A)** An ROC curve for predicting if an individual received the high or low nutritional supplement using all of the dried sweat mass normalized log_2_ fold change values. AUC indicates area under the curve. **(B)** A table of results from stepwise multiple linear regression modeling, utilizing bidirectional elimination, to predict performance metrics from using sweat metabolite quantities. The high and low nutritional supplement grouping variable was added as a confounding variable to account for differences in starting performance. The data illustrate the ability of relative sweat metabolite concentrations to predict if an individual was provided a high or low nutritional supplement with <20% false positivity. Furthermore, the relative sweat metabolite concentrations allow for significant prediction of several physical performance metrics.

The data presented in Figures 2, 3 suggest nutritional supplementation can influence sweat metabolomic results. To further illustrate that the sweat metabolome is impacted by nutritional supplementation, a receiver operating characteristic (ROC) curve was produced to identify if the metabolomics data (mass normalized log_2_ fold change values relative to week 1) can predict an individual's nutritional supplementation. Figure 4A shows the ROC curve utilizing all of the metabolomics data with an area under the curve (AUC of 0.82), suggesting with than 82% accuracy against false positives the sweat metabolomics data can predict if an individual ingests high or low dietary supplementation. When ROC curves are generated using only the data from the middle weeks (week 5, 6, or 7, AUC of 0.69) or the final week (week 12, AUC 0.81), the AUC values are less than the curve produced using all of the data points (Supplementary Data 11). These data provide further support for the hypothesis that nutritional intake can significantly impact the excreted sweat metabolome.

To determine the impact of nutritional supplementation on the relationships between sweat metabolites and performance, multiple linear regression modeling was applied to each individual performance metric. Delta performance, utilized to account for specific individual's change over time, between week 1 and 12 were used where applicable (Supplementary Data 1, Figure 4B). As significant difference in VO_2_ max was observed for the two groups, the supplement groups were added as a confounding variable within the analysis to account for the potential influence of baseline difference in performance (Supplementary Data 2). The results suggest three performance metrics: VO_2_ max, peak lower body Windgate, and abdominal circumference, have significant *p*-values (*p* < 0.05), showing that a linear relationship of metabolite values can significantly predict these metrics (Figure 4B). Collectively, the results suggest nutritional supplementation affects the excreted sweat metabolome, independent of dietary supplementation, and sweat metabolites can predict physical performance metrics.

## Discussion

As the biomarker discovery field expands into novel biosources, sweat has become an attractive non-invasive real time media for performance monitoring. As such, it is important to define new approaches for data normalization purposes. Previously, data was shown illustrating localized sweat rate normalization of metabolomics data caused greater dispersion among global metabolomics data while illustrating high intercorrelation among metabolite abundances when compared to non-normalized or gravimetric normalized data (Harshman et al., 2021). While the collection methodology to determine sweat rate outlined by Harshman et al. is advantageous when smaller volumes of sweat can be utilized for analysis, sufficient normalization approaches for larger quantities, such as those obtained from patches, have yet to be determined. Although historically gravimetric data normalization has been applied to bulk sweat collections like patches, gravimetric approaches are likely inaccurate (Patterson et al., 2000, 2002; Hayden et al., 2004; Morgan et al., 2004; Alvear-Ordenes et al., 2005; Buono et al., 2007; Meyer et al., 2007; Harshman et al., 2021) The results shown in Figure 2 indicate, similar to those using localized sweat rate, dried powder mass normalization caused a greater separation among the data when compared to the non-normalized/gravimetric normalization (Figures 2A,B) (Harshman et al., 2021). Additionally, as shown previously with localized sweat rate, there is a greater correlation among the metabolite abundances when normalized to the dried powder mass compared to the non-normalized/gravimetric normalized abundances (Figures 2C,D) (Harshman et al., 2021). Overall, these results suggest dried powder mass normalization of sweat metabolite values show similar trends as other sweat data normalization approaches (Harshman et al., 2021). These data suggest dried powder mass normalization is a viable option for data normalization collected from large volume sweat collection approaches.

The data presented in Figure 2B suggests that the low nutritional supplementation group had greater variability in their data, illustrated by a greater spread of the gray data points, compared to those taking the high nutritional supplement (black data points). It is reasonable to hypothesize this result is representative of a more complete total body nutrition within the high supplementation group compared to the low supplementation group. Furthermore, it is plausible that greater variability in the low nutritional group observed in Figure 2B is a result of participants relying more on their inconsistent individual diets to provide adequate nutrition rather than compensation by nutritional supplementation. Interestingly, Figure 3B illustrates the high nutritional supplementation group had a reduced log_2_ fold change relative to week 1 of the measured metabolites compared to the low nutrition group. Again, it is hypothesized that the reduction of the relative metabolite abundance within the high nutritional supplement are a result of stabile overall nutrition during the experiment while the low nutritional group was more variable and reliant on outside diet to provide nutrition. While merely hypotheses surrounding our data, the historical literature is inconsistent surrounding diet and sweat metabolite content. For instance, Hier et al. suggest diet has little immediate impact on amino acid content of whole body sweat, proposing a weak link between plasma and sweat, although only a short timeframe was evaluated (Heir et al., 1946) Conversely, Gitlitz et al. suggest amino acid content in sweat is a result of transfer from the intestinal fluid indicating a potential delayed link between the blood and sweat glands (Gitlitz et al., 1974). While plausible, these hypotheses must be further explored, in relation to blood level and potentially intestinal fluid levels, to fully understand the relationship between diet and sweat metabolites including amino acids.

Of the components contained in the high nutritional supplement and measured within sweat, only one compound, choline, was contained in both. Investigation of the choline data illustrated that no direct increase of choline was observed in the high nutritional supplement group suggesting an indirect mechanism for its presence in sweat. For example, the choline results show only a single sample (7.08 μM), among all the samples, was within the calibrated range (5–100 μM). These results suggest while choline was provided directly within the high nutritional supplement, a relative increase in choline abundance in sweat was not observed. Interestingly, these data are in line with previous results suggesting plasma choline levels do not change during training with or without choline supplementation (Spector et al., 1995). Although these data require further research, the results support the hypothesis that specific metabolites are introduced into sweat from many different potential sources (Baker and Wolfe, 2020).

While research linking diet and physical performance is well-established, examination of links between sweat small molecule metabolite abundance and human performance are less prevalent (See Purvis et al. (2013) and Baker (2019) for a review). Lactate has been the primary sweat metabolite, due to the link between blood lactate levels and muscle fatigue during exercise, investigated linking performance and sweat (Fellmann et al., 1983; Lamont, 1987; Pilardeau et al., 1988; Green et al., 2004). For instance, several studies have illustrated sweat lactate is inversely correlated with fitness level (Fellmann et al., 1983; Lamont, 1987; Pilardeau et al., 1988). Furthermore, Liappis et al. suggested that sweat amino acids are found in lower quantities in trained individuals compared to those untrained (Liappis et al., 1979). In this light, Figure 4B show results from a multiple linear regression modeling analysis to predict performance metrics using sweat metabolite abundance. These data illustrate an ability of semi-quantitative sweat metabolite values to predict performance metrics, such as VO_2_ max and peak lower body Windgate. Further investigation of the fitted regression coefficients for the significant overall models suggests features previously identified to correlate with performance metrics significantly contribute to overall models (Supplementary Data 12) (Harshman et al., 2021). For example, Harshman et al. suggested features tentatively identified as methionine and ornithine were correlated with VO_2_ max values (Harshman et al., 2021). Similarly, the multiple regression modeling within this study suggests both ornithine and methionine, significantly contribute to the prediction of VO_2_ max model. While it is currently unknown what underlying physiological and biochemical mechanisms may contribute to this observation, these data further support the growing evidence utilizing sweat as a biosource for performance estimation.

## Data Availability Statement

The datasets presented in this study can be found in online repositories. The names of the repository/repositories and accession number(s) can be found here: https://www.ebi.ac.uk/metabolights/, MTBLS2427.

## Ethics Statement

The studies involving human participants were reviewed and approved by Wright-Patterson Air Force Base Institutional Review Board. The patients/participants provided their written informed consent to participate in this study.

## Author Contributions

SH: conceptualization, methodology, investigation, formal analysis, writing original, review and editing, visualization, and supervision. AB: methodology, investigation, and writing – review and editing. CD: software, validation, formal analysis, visualization, writing original, and review and editing. RP: conceptualization, methodology, investigation, resources, supervision, and writing – review and editing. KS: methodology and investigation. NS: investigation and resources. MP: investigation, writing original, and review and editing. MO'C, NM, and JE: investigation and data curation. KB: investigation. AS and JM: supervision, project administration, and funding acquisition. All authors contributed to the article and approved the submitted version.

## Conflict of Interest

SH, AB, KS, NS, and MP are employed by company UES Inc. MO'C, NM, KB, and JE are employed by company InfoSciTex Corp. The authors declare that this study received funding from United States Air Force. The funder had the following involvement with the study: decision to publish. The remaining authors declare that the research was conducted in the absence of any commercial or financial relationships that could be construed as a potential conflict of interest.
