# Supplementary material for: The Impact of Nutritional Supplementation on Sweat Metabolomic Content: A Proof-of-Concept Study
Source: Front Chem. 2021 May 7;9:659583. doi: 10.3389/fchem.2021.659583 (PMC8138560; doi:10.3389/fchem.2021.659583)
Supplement: Supplementary file 1 [file Table_1.DOCX]

Supplemental Data

**SUPPLEMENTAL DATA 1:** A summary of the pre- and post-performance data. All delta values are post – pre. Capt.: Captain, SSgt.: Staff Sergeant, 2Lt.: Second Lieutenant, TSgt.: Tech Sergeant, SrA.: Senior Airman, A1C.: Airman 1^st^ Class, 1Lt.: First Lieutenant. Ab Cir: Abdominal Circumference, LBW: Lower Body Windgate, UBW: Upper Body Windgate, PP: Peak Power, AP: Average Power. Subjects 1-7 received the low nutritional supplement and subjects 8-13 received the high nutritional supplement.


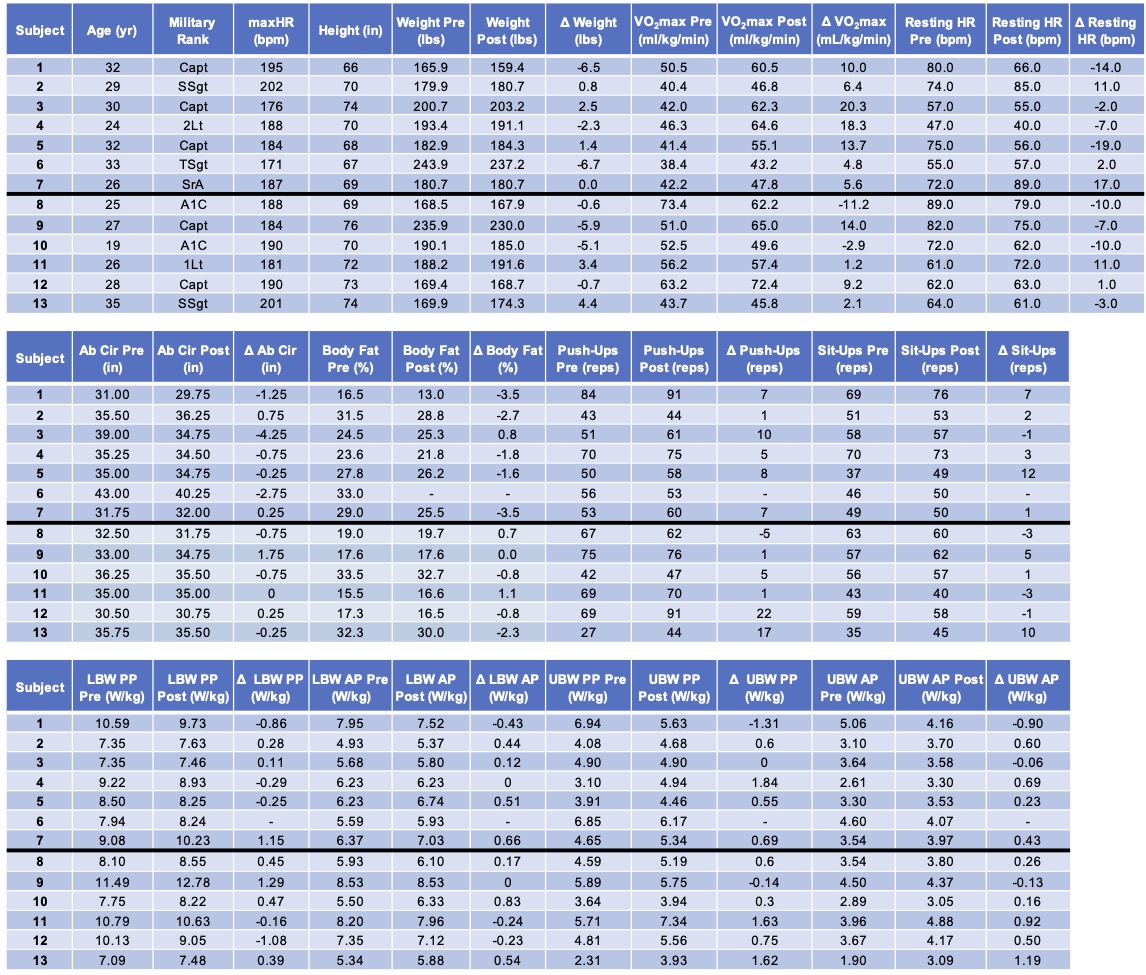


**SUPPLEMENTAL DATA 2:** A table summarizing the evaluation of the pre performance attributes among the two supplement groups, low and high. * indicates a significant difference between the two supplemental groups (p<0.05).

**­
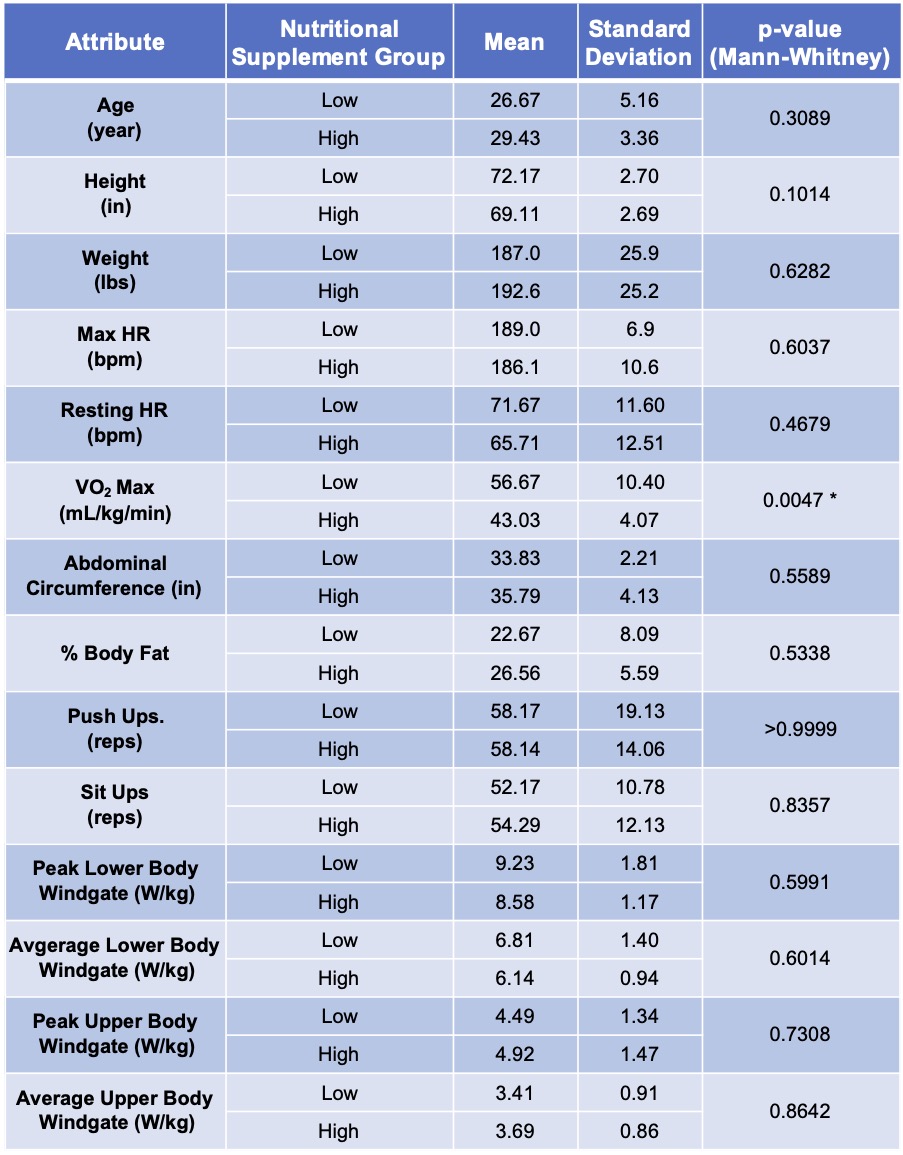
**

**SUPPLEMENTAL DATA 3: Left:** A summary of the exercise protocols, duration, and heart rate zones. **Right:** A summary of the exercise protocol, exercise performed, and the calculated training load. Training load is defined as “a numeric value of the approximate measure of carbohydrate and protein used as energy during training” by the Polar Team 2 Software (Polar Electro Inc., Lake Success, NY, USA). Exercises: T: Treadmill, E: Elliptical, B: Exercise Bike.

**
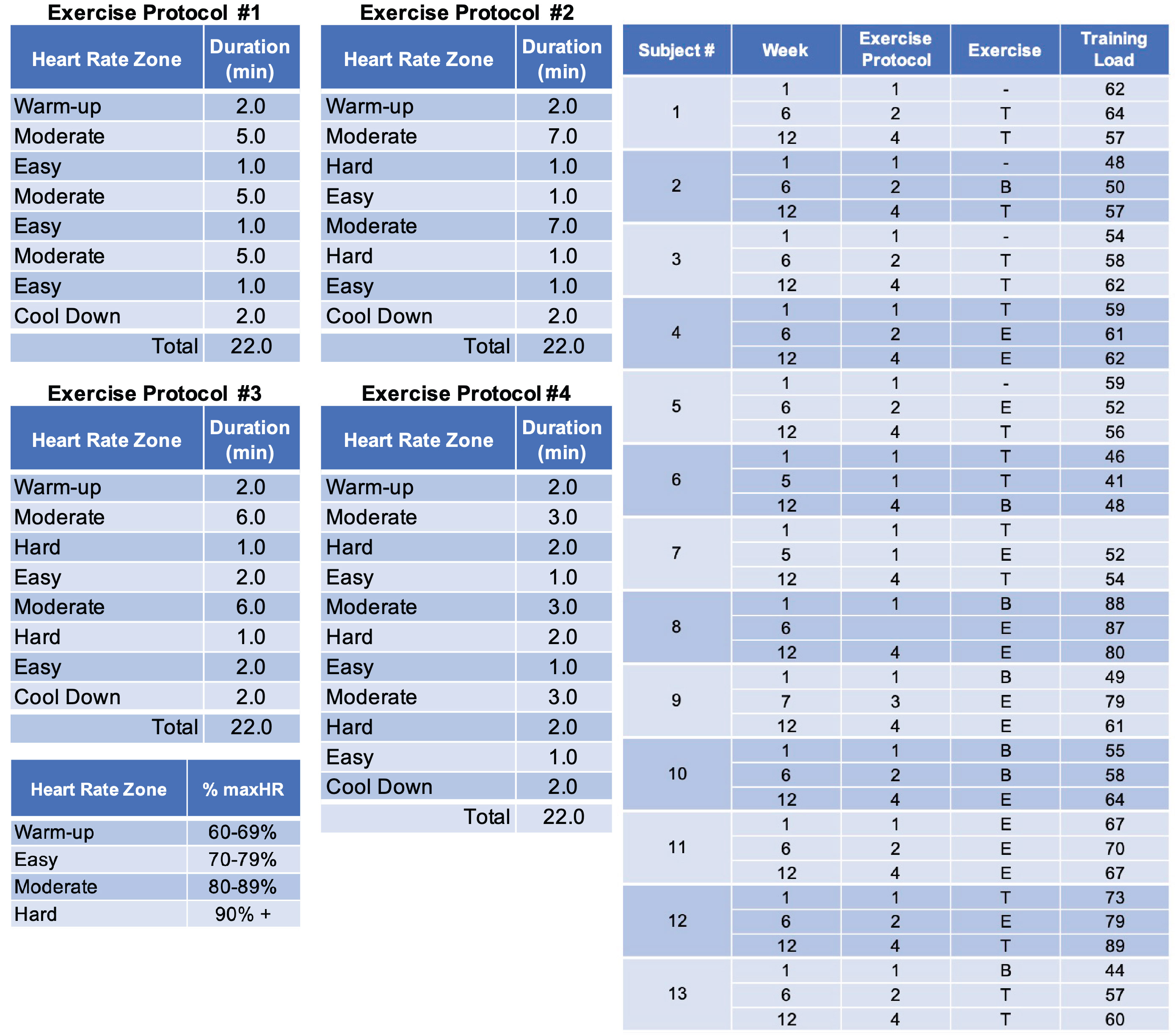
**

**SUPPLEMENTAL DATA 4:** A scatter plot of the exercise room temperature for each sweat sampling date. Error bars represent the 95% confidence interval about the mean.


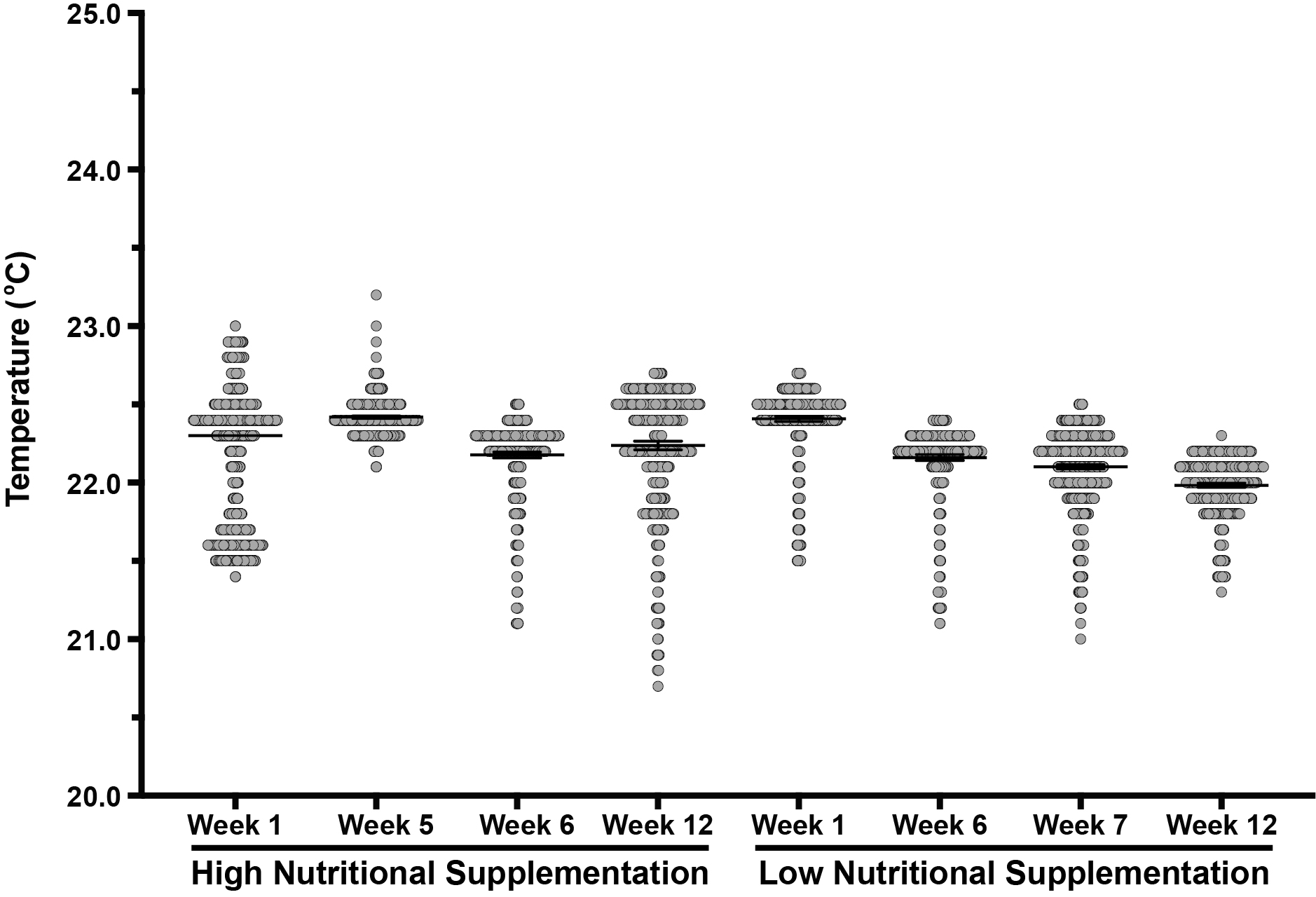


**SUPPLEMENTAL DATA 5:** A summary of each subject’s lyophilized sweat mass from each collection.


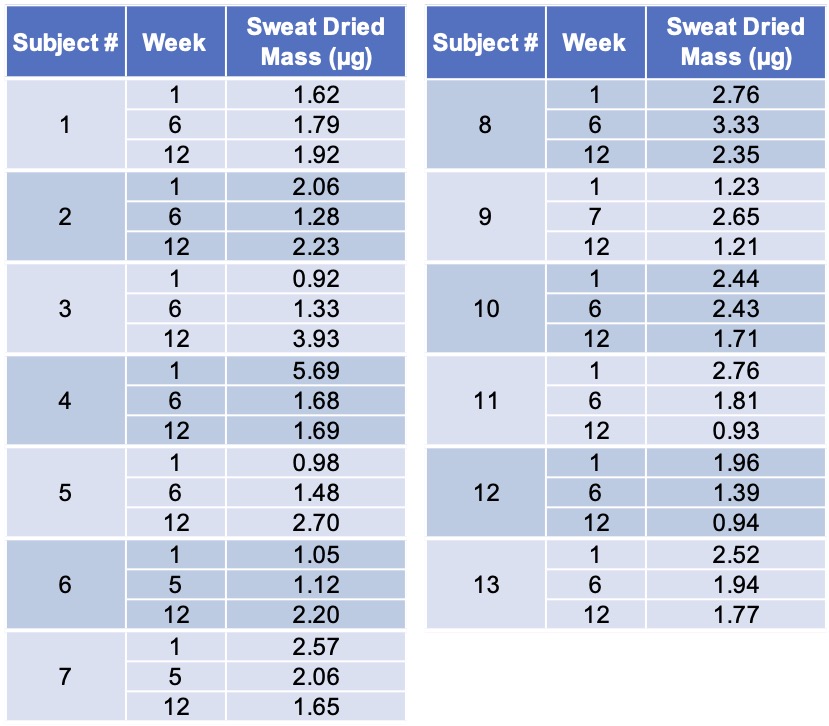


**SUPPLEMENTAL DATA 6:** A summary of the unlabeled and isotopically labeled standards used in the analysis.

**
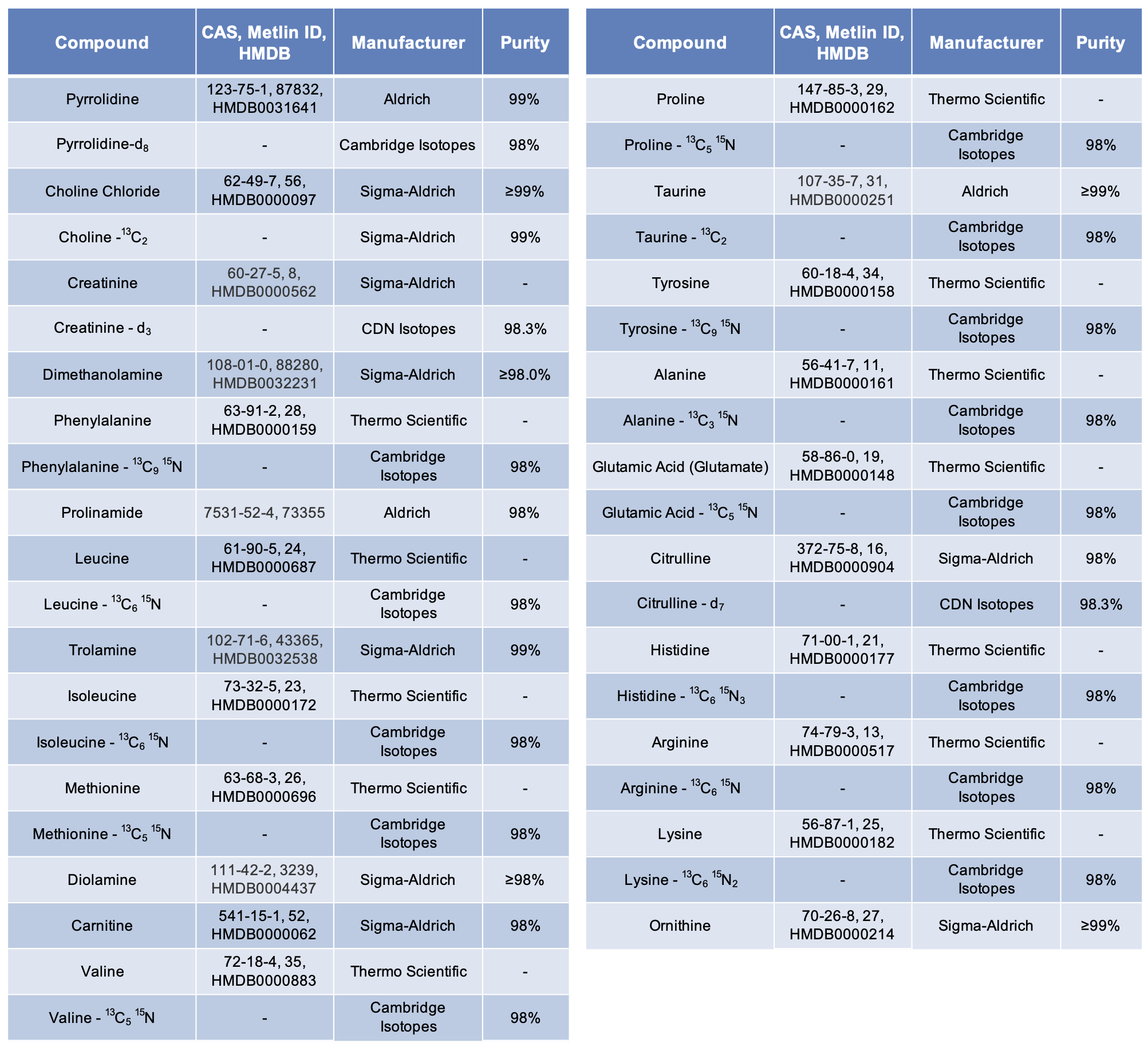
**

**SUPPLEMENTAL DATA 7:** A summary of the calibration curves generated for 23 sweat compounds. Q-ion: Quantitative ion.

**
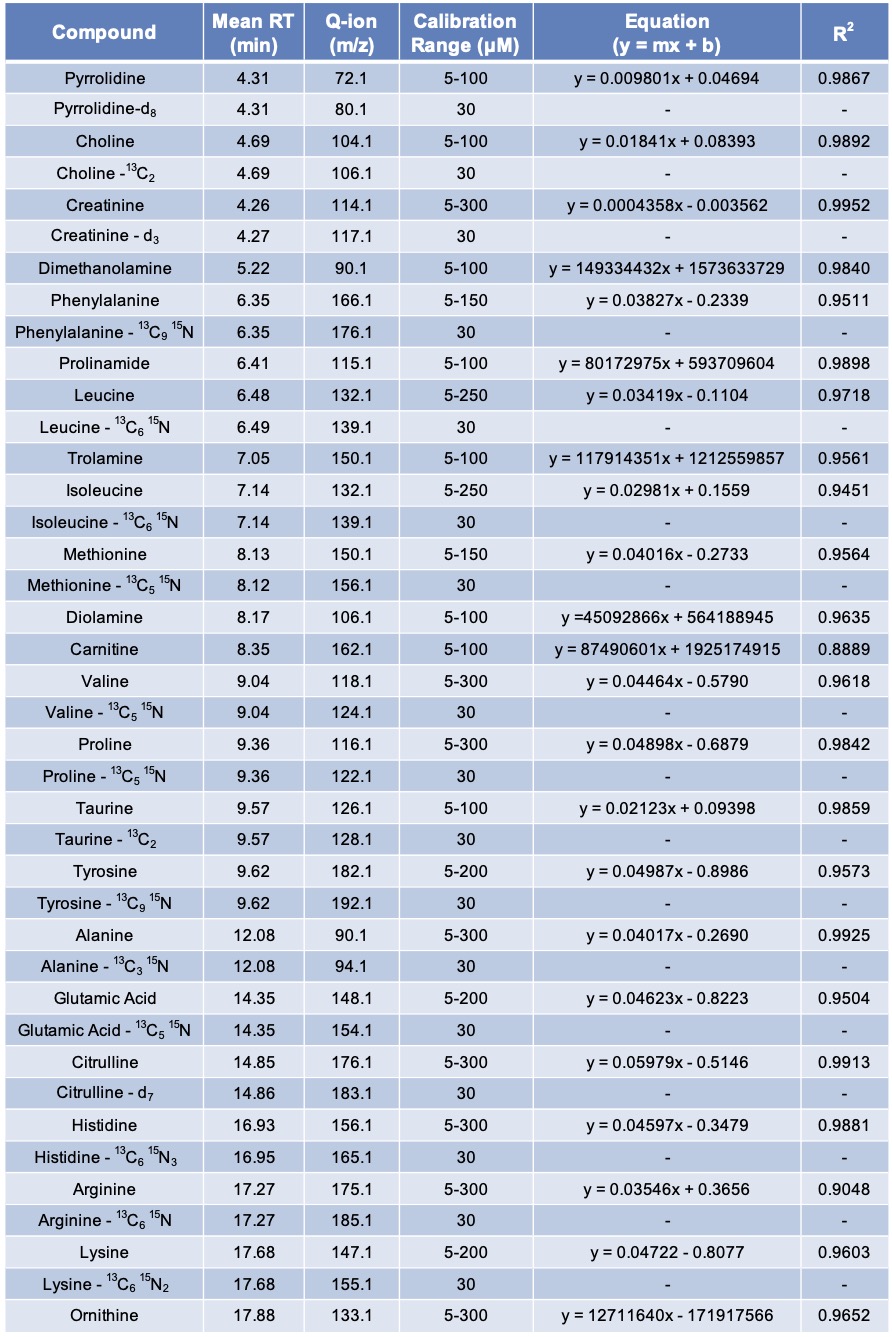
**

**SUPPLEMENTAL DATA 8:** A .xlsx file containing all processed data from the analysis.

**SUPPLEMENTAL DATA 9:** **A**) A summary of the calibrated range, mean sample retention time, and sample semi-quantitative concentration ranges **B)** A summary of the semi-quantitative results from the ­8 pooled sample injections. RT: retention time.

**
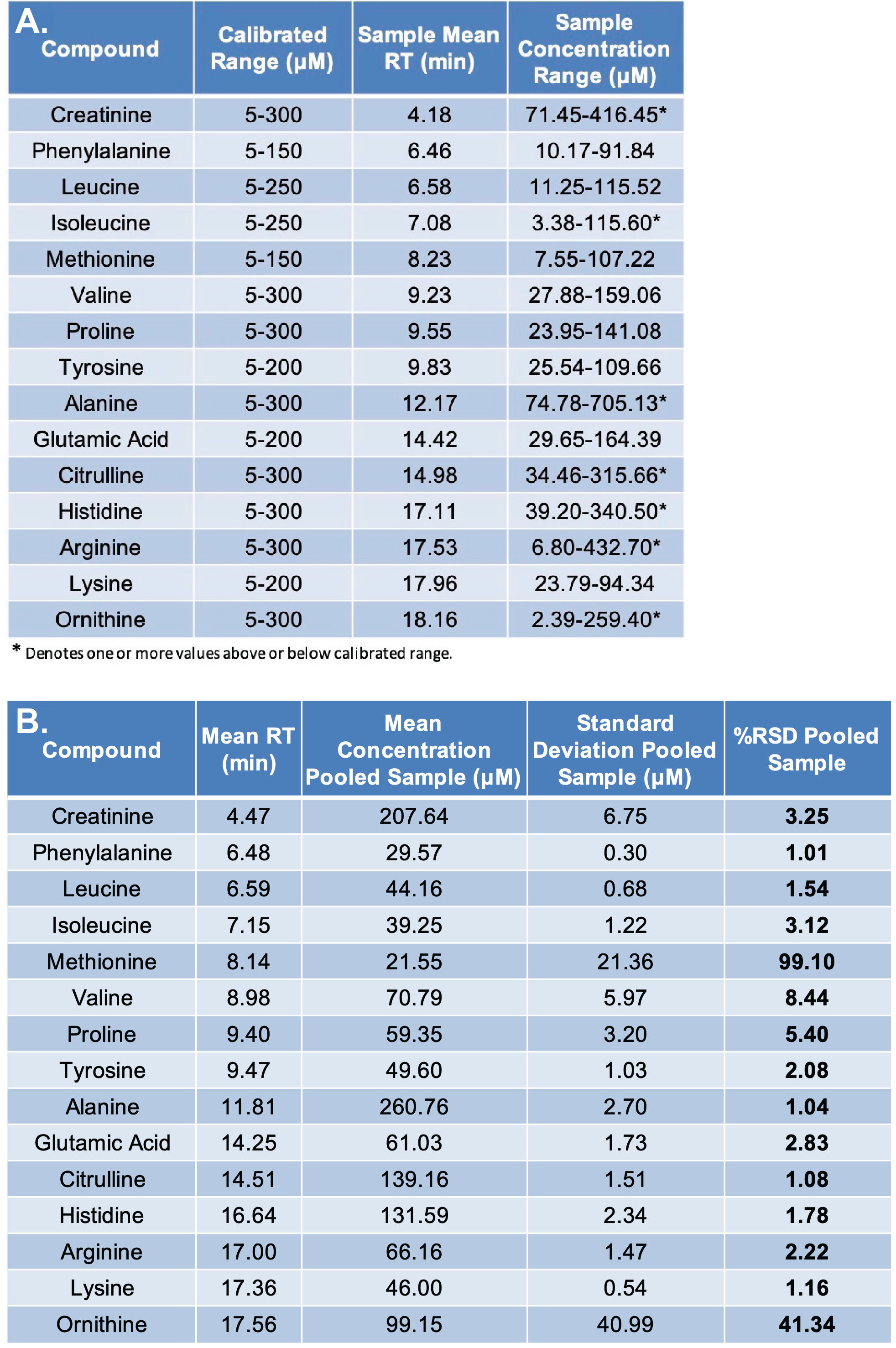
**

**SUPPLEMENTAL DATA 10:** The variable biplot (left) and the PCA loadings (right) for the metabolite semi-quantitative values.

**
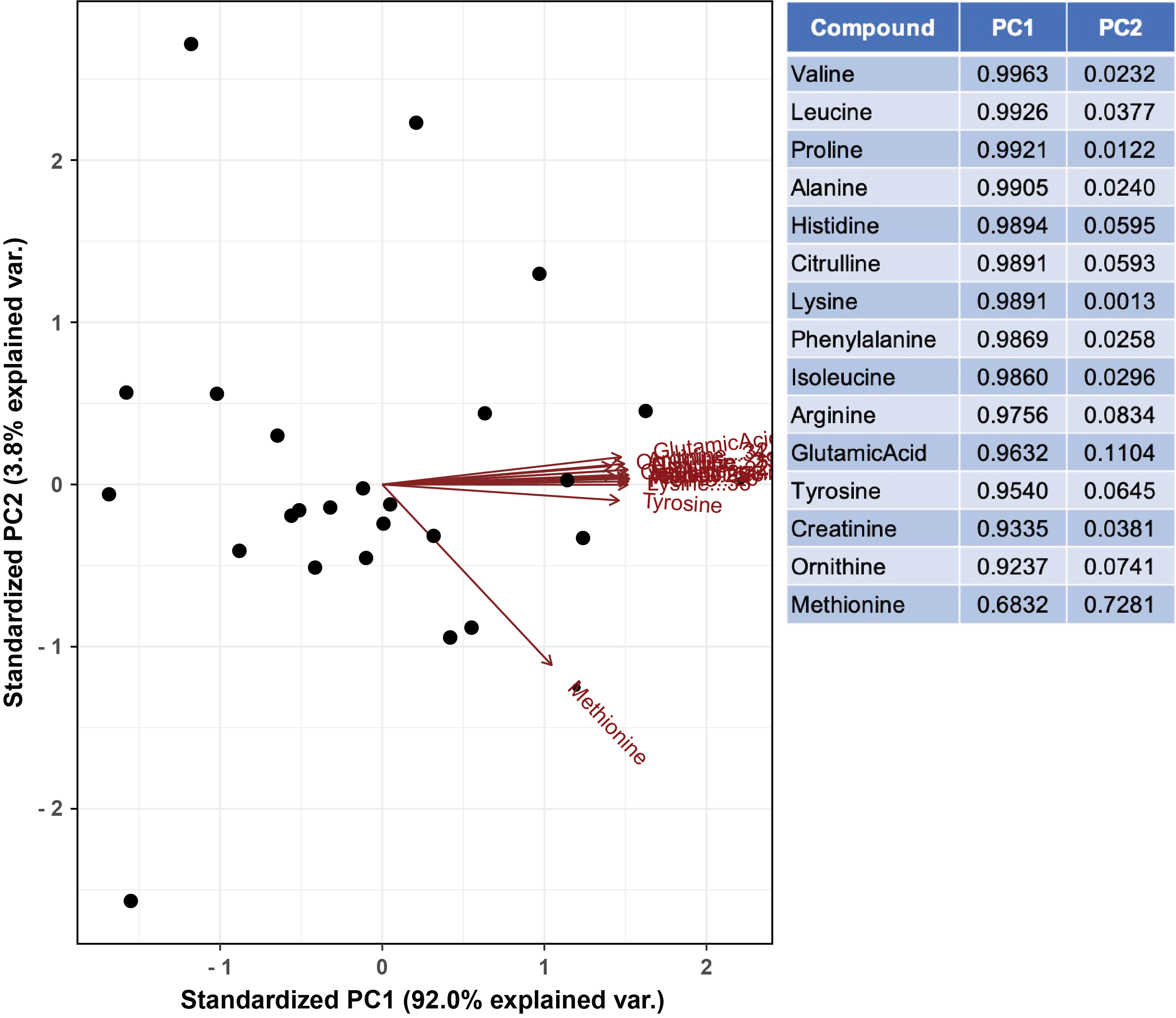
**

**SUPPLEMENTAL DATA 11:** (A) An ROC curve for predicting if an individual received the high or low nutritional supplement using only the middle weeks (week 5, 6, or 7) of the dried sweat mass normalized log_2_ fold change values to week 1. (B) An ROC curve for predicting if an individual received the high or low nutritional supplement using only the final weeks (week 12) of the dried sweat mass normalized log_2_ fold change values to week 1.

**
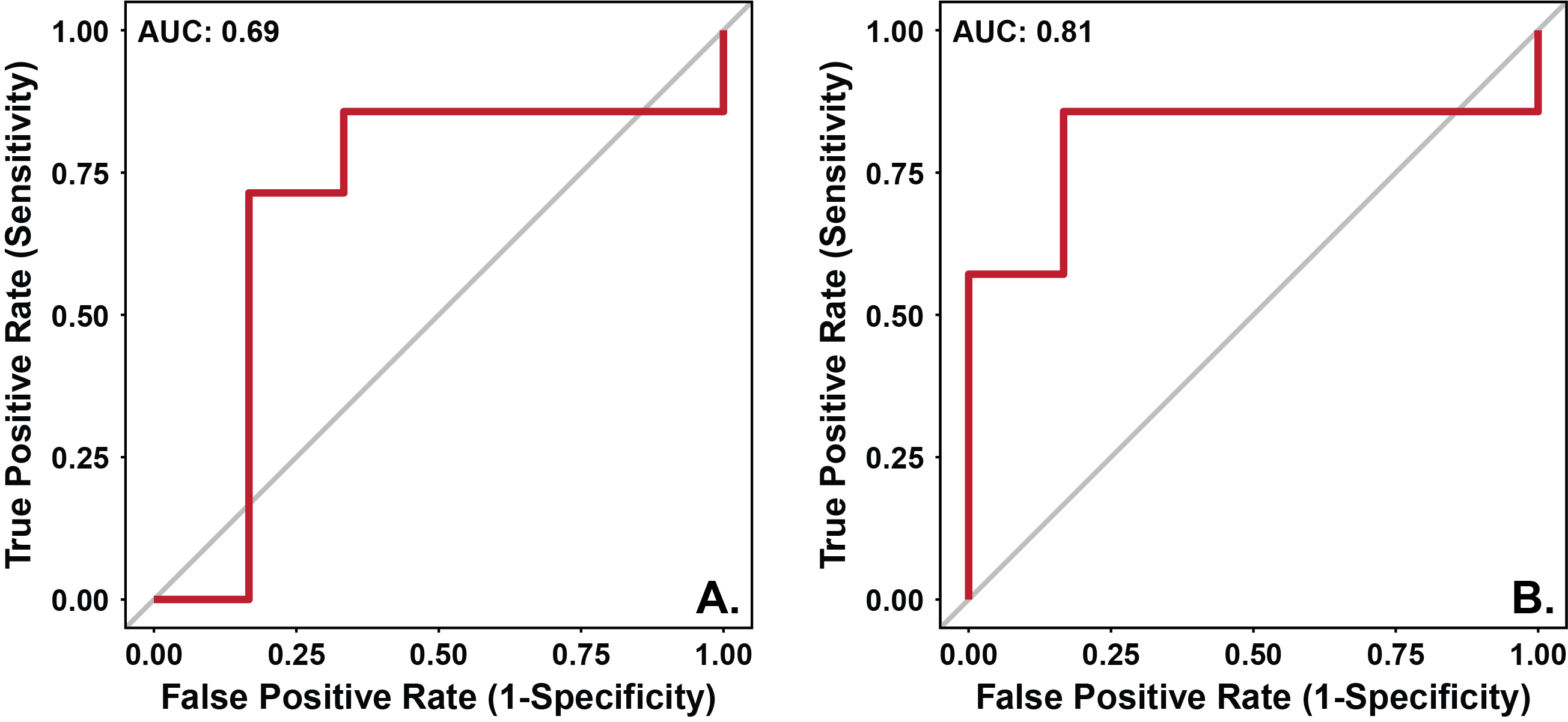
**

**SUPPLEMENTAL DATA 12:** A table summarizing the fitted regression coefficients and p-values for the independent variables or metabolites of the multiple regression models showing overall model significance.

**
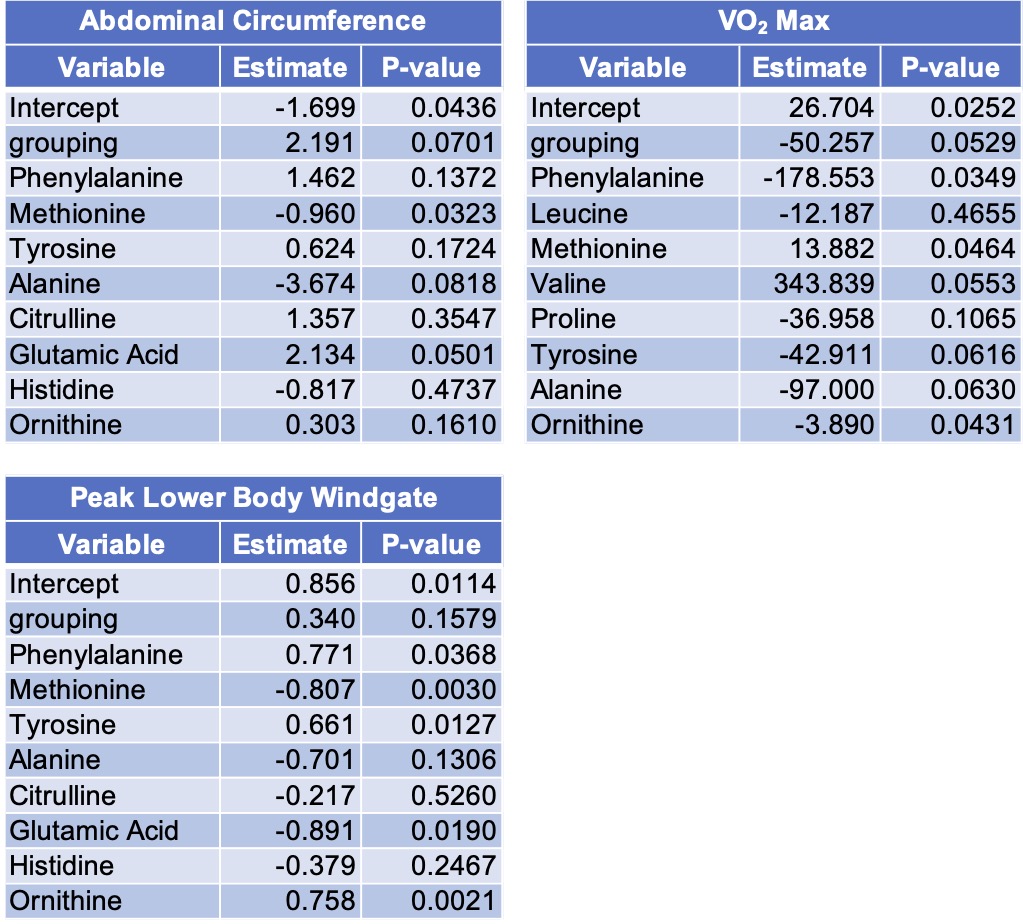
**
